# Degradation of Ciprofloxacin in Water by Magnetic-Graphene-Oxide-Activated Peroxymonosulfate

**DOI:** 10.3390/toxics11121016

**Published:** 2023-12-13

**Authors:** Xiaoping Wang, Yulan Li, Jiayuan Qin, Ping Pan, Tianqing Shao, Xue Long, Debin Jiang

**Affiliations:** 1 Chongqing Key Laboratory of Catalysis and Environmental New Material, College of Environment and Resources, Chongqing Technology and Business University, Chongqing 400067, China; liyulan201713@163.com (Y.L.); 18883248697@163.com (J.Q.); shao_tian_qing@163.com (T.S.); jansev1331@163.com (X.L.); jiangdebin@ctbu.edu.cn (D.J.); 2 Chongqing Ecological Environment Monitoring Center, No. 252, Qishan Road, Ranjiaba, Yubei District, Chongqing 401147, China; cqjczgb@126.com

**Keywords:** magnetic graphene oxide, heterogeneous activation, peroxymonosulfate activation, sulfate radical, ciprofloxacin

## Abstract

Antibiotics are extensively applied in the pharmaceutical industry, while posing a tremendous hazard to the ecosystem and human health. In this study, the degradation performance of ciprofloxacin (CIP), one of the typical contaminants of antibiotics, in an oxidation system of peroxymonosulfate (PMS) activated by magnetic graphene oxide (MGO) was investigated. The effects of the MGO dosage, PMS concentration and pH on the degradation of CIP were evaluated, and under the optimal treatment conditions, the CIP degradation rate was up to 96.5% with a TOC removal rate of 63.4%. A kinetic model of pseudo-secondary adsorption indicated that it involves an adsorption process with progressively intensified chemical reactions. Furthermore, the MGO exhibited excellent recyclability and stability, maintaining strong catalytic activity after three regenerative cycles, with a CIP removal rate of 87.0%. EPR and LC-MS experiments suggested that •OH and SO_4_^−^• generated in the MGO/PMS system served as the main reactants contributing to the decomposition of the CIP, whereby the CIP molecule was effectively destroyed to produce other organic intermediates. Results of this study indicate that organic pollutants in the aqueous environment can be effectively removed in the MGO/PMS system, in which MGO has excellent catalytic activity and stabilization for being recycled to avoid secondary pollution, with definite research value and application prospects in the field of water treatment.

## 1. Introduction

Quinolone antibiotics are a class of synthetic antibacterial drugs, which are widely used in health care and aquaculture due to the advantages of the antibacterial effect, structural simplicity and low price [1,2]. Ciprofloxacin (CIP) is a widely used quinolone antibiotic, primarily for the treatment of various bacterial infections. In recent years, CIP has been detected in surface water, groundwater, sewage treatment plants, medical wastewater, aquaculture, river sediments, etc. [3,4]. The complex structure and high antibacterial activity of CIP, as well as the difficulty of biodegradation and certain biological toxicity, make it difficult to be removed by conventional wastewater treatment processes [5]. Consequently, it is particularly crucial to develop an economical and efficient method for removing CIP in water.

A variety of methods are currently available for treating the CIP organic pollutants in an aqueous environment, including biological, physical and chemical methods [6,7,8]. Advanced oxidation technology based on a sulfate radical (SO_4_^−^•, oxidation potential = 2.5~3.1 V) generated by activation of persulfate has been developed in recent years for degradation of refractory organic pollutants in water. Compared with the hydroxyl radical (•OH, oxidation potential = 1.9~2.7 V) generated by catalytic hydrogen peroxide, the sulfate radical (SO_4_^−^•) exhibits the features of a long lifespan, broad spectrum of action, wide application range of pH and strong selectivity and can be generated by activation of peroxodisulfate (PDS) or peroxymonosulfate (PMS) in various methods [9,10]. PMS is a structurally asymmetric oxidant, which is easier to excite or activate than PDS, generally producing •OH and SO_4_^−^•with the activation of transition metals (homogeneous and heterogeneous) [11], ultraviolet light [12], ultrasound [13], heating [14], a carbon catalyst [15], etc. It has been demonstrated in abundant studies that the utilization of peroxymonosulfate advanced oxidation technology provides positive results for the treatment of CIP in wastewater [16,17,18,19].

Transition metal ion activation serves as the primary option for the homogeneous activation of PMS on account of its simplicity of operation, low cost and high removal efficiency. Nevertheless, the high dosage of transition metal ions leads to an increase in the amount of residual sludge, which is prone to cause secondary contamination [20]. In comparison, heterogeneous activated PMS provides the advantages of a wide pH range and high recovery rate of the catalyst. As Co has been identified to be the most effective activator for PMS [21], currently, it is preferred by many scholars regarding choosing multi-phase cobalt oxides for the activation of PMS. However, the leaching of cobalt poses a new health hazard, which can easily lead into cobalt toxicity when exposed to the environment containing cobalt for a long period of time [22]. Certainly, it has been found that the stability and catalytic properties of cobalt composites can be enhanced as a result of modifying the preparation method [23]. Graphene oxide (GO) [24], an oxidized derivative of graphene, is a novel carbon material. An abundant amount of oxygen-containing energy groups were found at both the basal surface and the edges of GO, allowing GO with a sufficiently many sorption sites, as well as being easily dispersed in water [25]. However, owing to the fact that GO separates poorly in water and is prone to cause new environmental problems, it is commonly used as a supporting material, attracting extensive attention on supporting metals and metal oxides [26]. The distinct characteristics of GO in terms of specific surface area size, oxygen-containing functional groups, adsorption capability and stability performance make GO an essential candidate for solid carrier materials in heterogeneous catalysts [25]. In addition, during compounding with metals, metal oxides, polymers and other materials, GO can effectively disperse the adhering materials and prevent agglomeration owing to its large specific surface area [27]. Several investigations revealed that GO, reduced graphene oxide and GO metal composites allowed the absorption and degradation of CIP through the activation of peroxomonosulfate [28,29].

In this paper, GO was prepared by the modified Hummers method [30] where there is an introduction of a significant number of oxygen-containing groups providing active sites for surface modification as well as a large specific surface area in the synthesis of GO-based materials [31,32]. Moreover, MGO composites were prepared with Fe_3_O_4_ particles and GO compounded by the ultrasonic chemical co-precipitation method. In this study, the feasibility of MGO-catalyzed PMS for the degradation of CIP was investigated. The effects of various influential factors (MGO content, PMS concentration, pH, etc.) on CIP removal were explored, the kinetic study of CIP removal was carried out and the possible degradation mechanisms and degradation pathways of CIP were proposed. A significant advantage lies in the superior catalytic activity and high stability of MGO composites, which can be recycled upon use, avoiding secondary pollution. It is expected that the outcomes of this study will offer a certain theoretical basis and fundamental support for the practical implementation of MGO/PMS-based advanced oxidation technology in the treatment of antibiotic wastewater.

## 2. Materials and Methods

### 2.1. Reagents

The reagents mainly consisted of natural graphite (99%), sulfuric acid (H_2_SO_4_, 98%), potassium permanganate (KMnO_4_), hydrogen peroxide (H_2_O_2_, 30%), ferric chloride hexahydrate (FeCl_3_·6H_2_O), ferrous sulfate heptahydrate (FeSO_4_·7H_2_O), anhydrous ethanol (CH_3_CH_2_OH, 99.5%), potassium hydrogen persulfate (KHSO_5_). Chromatographic-grade acetonitrile and methanol were used as the mobile phase, and ciprofloxacin (CIP) was purchased from MACKLIN (Shanghai, China). All reagents with which the study was carried out were of an analytically pure grade without specific instructions. In addition, all solutions used for the experiments as prepared with deionised water.

### 2.2. Synthesis of MGO Catalysts

**Figure 1:**
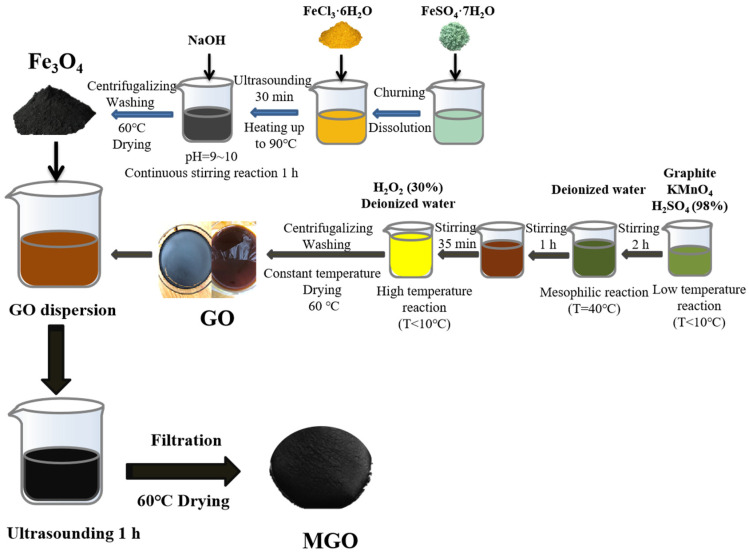
The fabrication process of MGO.

Figure 1 shows the preparation process for the synthesis of MGO using ultrasonic chemical precipitation. Firstly, 1.000 g of GO was transferred into 100 mL of deionized water. After that, it was sonicated for 30 min, and 1.000 g of Fe_3_O_4_ was added and mixed well and continued to be sonicated for 1 h. Finally, the filtered reactants were collected and then dried in an oven (60 °C), resulting in MGO.

### 2.3. Catalysts’ Characterization

The physical properties of the materials prepared in the study were mainly determined with a scanning electron microscope (SEM, thermo scientific Apreo 2C, ThermoFisher Scientific, Waltham, MA, USA), transmission electron microscopy (TEM, FEI Talos F200X, ThermoFisherscientifi USA) and a specific surface and porosity analyzer (BET, ASAP 2020, Mike instruments, Norcross, GA, USA). The materials in anhydrous ethanol were dispersed using ultrasonication, and scanned with an SEM and TEM, respectively, from which the morphological information of the activated materials was clarified, structural defects were analyzed and the composite of the materials was determined, whereas the specific surface area and the pore structure were identified with BET.

In addition, X-ray diffraction (XRD, XRD-D8, Bruker, Karlsruhe, Baden Wurttemberg, Germany), Fourier transform infrared (FT-IR, IR Prestige-21, Shimazu, Japan) and X-ray photoelectron spectroscopy (XPS, ESCALAB Xi+, ThermoFisher Scientific, USA) were employed to analyze the crystal structure and chemical characteristics of the activated materials. The powdered materials were pressed into flakes, and then XRD was employed to identify the crystal structure and phase transition, with the scanning range of 0~80°, the scanning speed of 5°/min and the temperature maintained at 25 °C. Similarly, the materials in powder were homogeneously mixed with potassium bromide in the ratio of 1:100, which was pressed and scanned for the infrared absorption spectrum to observe the types of functional groups contained in the materials. And finally, XPS was performed to test the whole and fractional spectra of the materials, so as to analyze the elemental composition of the samples, with the AI target as the test target.

### 2.4. Degradation Experiment of CIP

The aqueous solution of CIP (20 mg/L) configured in the experiment was stored protected from light and used up within 1 week at a time, for a study on the degradation of CIP by MGO-composite-activated PMS, in which the CIP removal was used as a criterion for the catalytic performance of the material. The specific experimental steps were as follows: the activators (GO or MGO) and PMS with a certain concentration were added to a conical flask containing 100 mL of 20 mg·L^−1^ CIP-simulated wastewater, and then placed in a thermostatic culture oscillator (ZWYR-240, Shanghai Zhicheng Analytical Instrument Manufacturing Co., Ltd., Shanghai, China) to perform the oscillation reaction for 180 min at 25 °C and a rotational speed of 150 rpm. In addition, the effects of the initial pH of the CIP solution (3, 5, 7, 9, 11), PMS concentration (0.5, 1.0, 1.5, 2.0, 3.0 g/L) and catalyst content (0.5, 1.0, 1.5, 2.0, 3.0 g/L) on the removal efficiency of CIP were investigated.

In all cases, it was possible to repeat all experiments three times under the same conditions to facilitate the subsequent error analysis and calculations, and the removal efficiency of CIP was calculated by Formula (1):

(1) $$W=\frac{C_{0}-C_{t}}{C_{0}}\times 100\%$$

where *W* is the removal efficiency (%), and *C*_0_ and *C*_t_ denote the initial concentration of CIP and the concentration (mg·L^−1^) of CIP at time t, respectively.

### 2.5. Samples’ Analysis

The full wave of the CIP standard solution was scanned using a UV spectrophotometer (UV1102II, Shanghai, China), and 277 nm as a characteristic peak of CIP was determined. The concentration of CIP was detected by high-performance liquid chromatography (LC, LC-100 plus, Shanghai, China) with a Pronaos EP-C18 column (4.6 × 260 mm, 5 µm) and a UV detection wavelength of 277 nm. The mobile phase consisted of 0.1% formic acid and acetonitrile in a volume ratio of 80:20, which flowed for 0.30 mL·min^−1^, and the injection volume was 20 µL.

The main active species in the CIP degradation by MGO-composite-activated PMS were detected with electron paramagnetic resonance (EPR, EMX nano, Bruker, Karlsruhe, Germany), in which DMPO acted as a free radical trapping agent. The mineralization rate of the reaction solution was measured with a total organic carbon analyzer (TOC, TOC-L CPH/CPN, Shimadzu, Kyoto, Japan), and the TOC removal rate as the basis for the oxidative degradation of CIP.

The intermediates of the CIP degradation were analyzed by liquid chromatography–mass spectrometry (LC-MS, Agilent LC1290-QQQ-6470, City of Santa Clara, CA, USA) with an ACE, UltraCore 2.5 µm C18 (2.1 × 75 mm) column of 35 °C. In addition, the mobile phase was acetonitrile and formic acid (0.1%) in a flow rate of 0.40 mL·min^−1^. The ESI was performed in the positive ionization mode and the scanning range of 200–400 *m*/*z*, as well as the mass spectrometry analysis being conducted in a gradient elution mode.

## 3. Results and Discussion

### 3.1. Characterization of Catalysts

#### 3.1.1. Analysis of Surface Physical Structure

**Figure 2:**
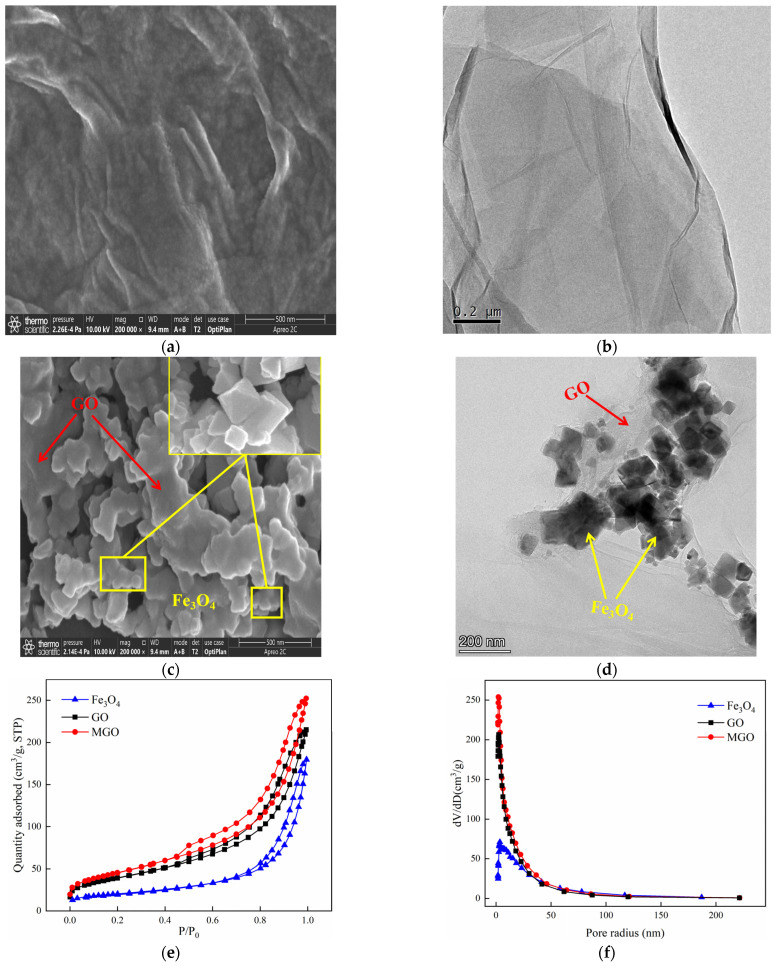
SEM of GO ((**a**): 500 nm) and MGO ((**c**): 500 nm); TEM of GO ((**b**): 200 nm) and MGO ((**d**): 200 nm); N_2_ absorption–desorption isotherms (**e**) of GO, Fe_3_O_4_ and MGO; and the pore–size distribution curves (**f**) of GO, Fe_3_O_4_ and MGO.

The SEM and TEM characterizations of GO and MGO materials were shown in Figure 2. As from Figure 2a,b, GO exhibited a translucent thin film sheet structure with a flat and smooth surface, visible wrinkles toward the edges of the sheet layer, as well as a low degree of polymerization among the sheets. Such a special structure effectively increased the specific surface area of GO, providing sufficient adsorption sites. At the same time, in the process of the powerful oxidation of GO, an abundant amount of oxygen-containing functional groups were inserted between the lamellae, with additional defects across the GO layers and expanded structural irregularities, resulting in the dispersion of GO in the solution, which facilitated the preparation of the composite materials. The SEM images of MGO are shown in Figure 2c, from which a large variety of Fe_3_O_4_ particles were well distributed on the surface of the MGO material. Additionally, the TEM image (Figure 2d) further demonstrated that the MGO material was successfully prepared by Fe_3_O_4_ particles and GO. Nano-Fe_3_O_4_ particles had a prismatic cone shape. By chemical precipitation, MGO uniformly loaded Fe_3_O_4_ particles between the surface and the sheet while retaining the original structure of GO.

The specific surface area is considered as one of the significant parameters to characterize the adsorption properties of the materials, whereas the pore size distribution may affect the catalytic function to which the materials are subjected. The N_2_ adsorption–desorption experiments of GO, Fe_3_O_4_ and MGO materials were performed so that the information of adsorption isotherms and pore size distribution of the materials were obtained. Figure 2e shows that the adsorption isotherms of GO, Fe_3_O_4_ and MGO were all type IV isotherms and presented an adsorption hysteresis phenomenon with an H3-type hysteresis loop, indicating that GO, Fe_3_O_4_ and MGO were all pore structures of layered materials. As shown in Figure 2f, the pore size of Fe_3_O_4_ was mainly in the range of 3–200 nm, and the void sizes of GO and MGO to be distributed were mainly between 3 and 100 nm. Table 1 illustrates that the specific surface areas of Fe_3_O_4_, GO and MGO were 70, 140 and 163 m^2^/g, respectively. Compared with Fe_3_O_4_ and GO, the specific surface area and pore volume of MGO composites increased, while the pore size decreased. Possibly, on account of the fact that the crystallinity of the sample reduced by the continuous ultrasonic reaction in the composite process of the material, where small molecules might be released from the GO carbon skeleton, generating a new pore structure, thus the specific surface area and void volume increased. However, the massive accumulation of Fe_3_O_4_ particles would lead to the reduction in pore size.

#### 3.1.2. Analysis of Crystal Structure and Chemical Characteristics

**Figure 3:**
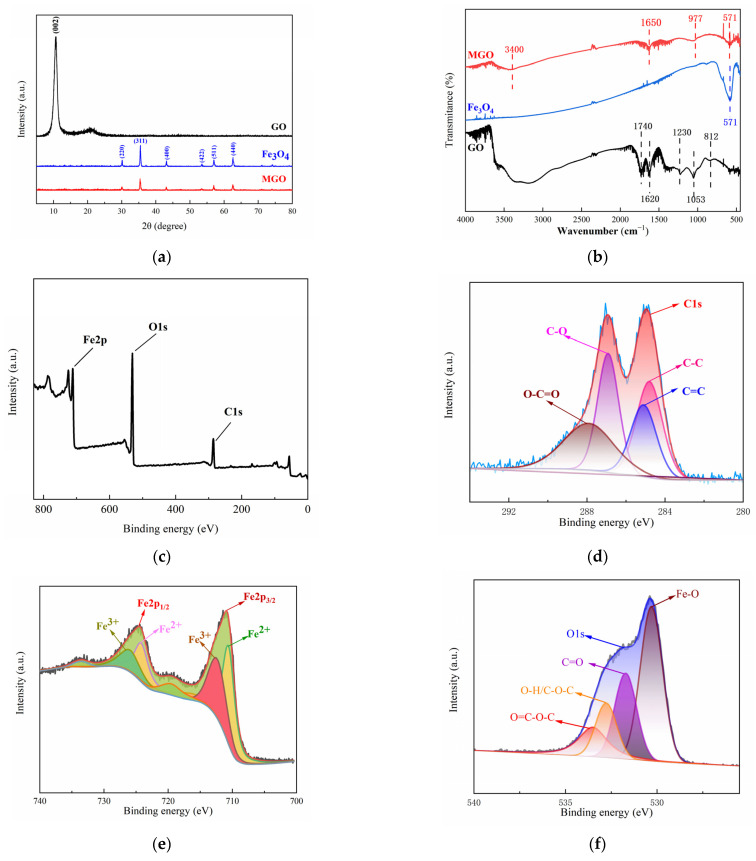
XRD patterns (**a**) and FT–IR spectra (**b**) of GO, Fe_3_O_4_ and MGO, and XPS spectra of MGO (**c**), C1s (**d**), Fe2p (**e**) and O1s (**f**).

The crystal structure characteristics of GO, Fe_3_O_4_ and MGO were analyzed by XRD patterns at the range of 0–80° (2θ). In Figure 3a, the diffraction pattern of GO showed an obvious diffraction peak at 2θ = 10.8°, corresponding to the XRD peak of the standard GO nanosheet (001), and the absence of the characteristic diffraction peak of graphite, indicating that graphite has been successfully oxidized to make GO. In addition, it was observed that significant characteristic peaks appeared in the XRD spectra of Fe_3_O_4_ at 2θ of 30.10°, 35.42°, 43.05°, 53.39°, 56.94° and 62.52°, which correspond to the diffraction peaks (220), (311), (400), (422), (511) and (440) for the pure cubic-spinel structure of Fe_3_O_4_ (Fe_3_O_4_ standard card PDF#196-0629), respectively. The diffraction peaks in the XRD spectra of MGO were identical to that of Fe_3_O_4_ and without the appearance of new diffraction peaks, demonstrating that an MGO composite material was successfully prepared. However, the intensity of the diffraction peaks with MGO were weaker than that of Fe_3_O_4_, which might be due to other lattice variations occurring within the compounding process, causing its lower average grain size and poorer crystallinity. In addition, no significant GO diffraction peak appeared at 2θ = 10.8°, which illustrated that the GO stacking in the composite presented a disordered state.

Figure 3b showed the infrared spectrograms of GO, Fe_3_O_4_ and MGO for analyzing the variations observed among the functional groups. Comparing the infrared spectrum of GO and MGO, it could be seen that several characteristic peaks similar to GO were observed in MGO, yet the stretching vibration peaks containing C=O and C-O were significantly shifted (1740 cm^−1^→1650 cm^−1^, 1053 cm^−1^→977 cm^−1^), with significantly weakened absorption peaks, which probably occurred as a result of the group change in the process of the material composite. Beyond that, the characteristic peak of Fe_3_O_4_ could be clearly observed at 571 cm^−1^, as a stretching vibration of the Fe-O bond, indicating that Fe_3_O_4_ particles were effectively introduced into the surface and lamellae of GO. The above FT-IR analysis results demonstrated the successful fabrication of Fe_3_O_4_-GO(MGO) composites, which contained an abundance of oxygen-containing functional groups.

The elemental composition and valence distribution of the MGO composites were characterized using X-ray photoelectron spectroscopy (XPS). The XPS whole spectrum (Figure 3c) revealed that MGO primarily consisted of just three elements, including C, O and Fe, with elemental percentages corresponding to 35.60%, 46.82% and 17.58%, respectively, as shown in Table 2. With the peak points observed at 284.0–288.0 eV, 710.0–725.5 eV and 531.0 eV for C1s, Fe2p and O1s, respectively [33,34,35], this suggested that Fe_3_O_4_ and GO compounded successfully.

The characteristic peaks of C1s (Figure 3d) were mainly distributed in the range of 284.0 to 288.0 eV, in which the peaks at 284.2, 285.0, 287.0 and 288.0 eV were identified as C-C, C=C, C-O and O-C=O, respectively. This indicated that the MGO composite material included chemical components such as cyclic hydrocarbons and olefins, and carboxyl groups and hydroxyl groups were contained on the surface, suggesting that MGO had a relatively complete structure. The fitting of the main peaks of Fe2p (Figure 3e) revealed that the characteristic peaks of Fe2p_1/2_ and Fe2p_2/3_ were 724.1 eV and 710.1 eV, respectively, with a difference in binding energies of approximately 14 ev. In this case, the Fe^2+^ peaks were located at 710.5 eV and 722.6 eV, whereas that of 711.0 eV and 725.3 eV belonged to the Fe^3+^ [36]. This indicated that the Fe element in the MGO composite mainly existed in the form of Fe_3_O_4_. Three relatively distinct peaks were observed in the energy spectrum of O1s (Figure 3f), in which the peaks around 530.0 eV and 532.8 eV corresponded to Fe-O in Fe_3_O_4_ and hydroxyl oxygen (O-H) on the surface of the material, respectively, as well as the characteristic peak at 530.8 eV belonging to carboxyl oxygen or carbonyl oxygen (C=O) [33,34,37]. Simultaneously, the large energy spectrum area could demonstrate that the MGO composites contained abundant carboxyl and hydroxyl functional groups, manifesting the structural integrity of the material, which was conducive to the adsorption and catalytic degradation of pollutants.

### 3.2. Degradation of CIP by MGO-Activated PMS

#### 3.2.1. Analysis of Crystal Structure and Chemical Characteristics

**Figure 4:**
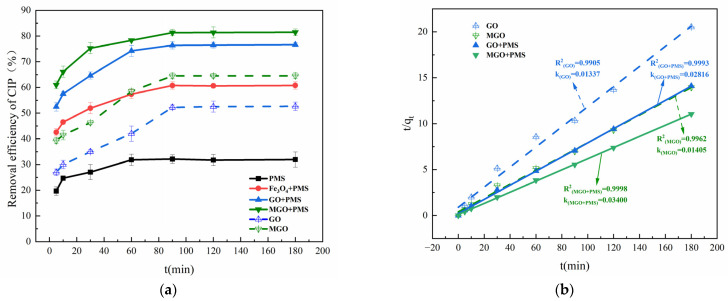
Removal of CIP in PMS alone, Fe_3_O_4_/PMS, GO/PMS and MGO/PMS (**a**). Pseudo-second-order adsorption kinetic model (**b**). Experimental conditions: [CIP] = 20 mg/L, [PMS] = 1.0 g/L, [Fe_3_O_4_, GO, MGO] = 0.5 g/L, T = 25 °C.

The removal effects of Fe_3_O_4_, GO and MGO-activated PMS on CIP are compared in Figure 4. As shown in Figure 4a, with PMS alone, only a 31.74% removal of CIP was achieved after 120 min of treatment, which was much lower than that in the presence of Fe_3_O_4_, GO or MGO. This indicated that Fe_3_O_4_, GO and MGO all have a certain activation effect on PMS, improving the CIP removal rate to 60.61%, 76.47% and 81.39% within 120 min, respectively.

In the Fe_3_O_4_/PMS system, for one thing, Fe_3_O_4_ nanoparticles adsorbed a certain concentration of CIP owing to the large specific surface area. For another, SO_4_^−^• generated from PMS activated by Fe^2+^ on the surface of Fe_3_O_4_ nanoparticles had a powerful oxidation effect and could remove CIP in water as shown in Reaction (2) [38,39]. However, it is possible that the specific surface area, the stability of dispersion and the catalytic activity of the catalysts may be decreased by the ease of aggregation of Fe_3_O_4_ nanoparticles in the solution [40].

(2) $$Fe^{2+}+HSO_{5}{}^{-}\to SO_{4}{}^{-}•+Fe^{3+}+OH^{-}$$

Several studies demonstrated that carbon-based materials could also activate PMS [41,42,43]. Gao et al. [44] reviewed the study on the degradation of antibiotic contaminants by activation of PMS with different carbon materials (activated carbon, biochar, carbon nanotube, other carbon materials or doped-carbon materials, etc.) and found that the majority of carbon materials with a low cost and chemical stability were commonly applied to activate PMS to produce active species, but with a low activation efficiency. In the GO/PMS system, •OH and SO_4_^−^• were generated from the activation of PMS with the abundance of oxygen-containing functional groups contained in GO. In addition, GO performed the role of a bridge of electron transfer to transfer electrons from organic compounds to PMS [13,45], with the following reactions, Reactions (3) and (4):

(3) $$HSO_{5}{}^{-}+e\to SO_{4}{}^{-}•+OH^{-}$$

(4) $$HSO_{5}{}^{-}+e\to SO_{4}{}^{2-}+•OH$$

As compared with the other two catalysts (Fe_3_O_4_ and GO), MGO displayed a better catalytic activation effect, which lead to a 80% removal of CIP within 120 min. By contrast, with the absence of PMS, the removal of CIP was just about 64% for the same reaction time by adsorption of MGO alone, which accounts for the synergistic catalytic effect of Fe_3_O_4_ and GO on PMS.

Kinetic investigations of the above reaction systems revealed that none of them conformed to the degradation kinetic model (Figure S1a,b), nor to the pseudo-first-order adsorption kinetic model (Figure S1c). The adsorption kinetic studies of GO, MGO, GO/PMS and MGO/PMS are presented in Figure 4b, and the linear correlation coefficients R^2^ = 0.9905, 0.9962, 0.9993 and 0.9998, respectively, which reached a significant level, with the adsorption rate constants k being 0.01337, 0.01405, 0.02816 and 0.03400 mg·(g·min)^−1^, approximately, demonstrating that the pseudo-second-order adsorption kinetic model better proportioned the dynamic process of CIP removal from the aqueous solution by GO, MGO, GO/PMS and MGO/PMS. As a heterogeneous reaction, the removal of CIP by MGO/PMS occurred as an adsorption process with a progressively enhanced chemical reaction, in no way suggesting that the removal was realized exclusively by adsorption of MGO alone. Actually, the degradation of CIP by MGO-activated PMS is a reaction process involving adsorption and degradation simultaneously, not a singular adsorption or degradation reaction. The degradation of CIP occurred while PMS and CIP were adsorbed onto the surface of MGO, in which the consumed PMS and the degraded CIP contributed new adsorption sites for MGO, leading to the circular reaction with adsorption and degradation being performed again. Enhanced adsorption capacity as indicated by an increase in the value of the adsorption rate constant k was not caused by improving the adsorption capacity of the MGO material itself; on the contrary, it is attributed to the fact that the oxidation reaction occurring on the MGO surface intensified the adsorption reaction processes of MGO.

#### 3.2.2. Effects of Key Factors on CIP Degradation

**Figure 5:**
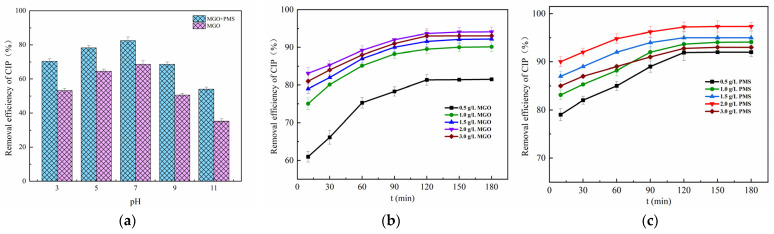
Effect of initial pH of solution (**a**), MGO dosage (**b**) and PMS concentration (**c**). Experimental conditions: [CIP] = 20 mg/L, T = 25 °C.

The initial pH effect of the solution between 3 and 11 on CIP adsorption and degradation is explored in Figure 5a. As can be seen, the adsorption of CIP showed to be weaker than the degradation reaction of CIP. The neutral conditions were favorable for the adsorption and degradation of CIP, in which case the CIP adsorbed and degraded by MGO was 64.53% and 82.46%, respectively, significantly higher than that of the acidic and alkaline conditions.

Typically, the initial pH of the solution would affect the existence form of PMS, the species and presence of free radicals as well as the process of the charge transferred [46], in which PMS was mainly in the form of HSO_5_^−^ in acidic and neutral conditions, while existing primarily as SO_5_^2−^ in the alkaline condition [47]. The more acidic the solution, the more prone HSO_5_^−^ was to form •OH with weaker oxidation capacity than SO_4_^−^•, which inhibited the degradation reaction of CIP. Meanwhile, the CIP, which was mainly in the form of cations in acidic conditions, repelled with the positive charges on the MGO surface, diminishing the electrostatic binding force between CIP and MGO, resulting in weakened adsorption of CIP. It was also found that the removal of CIP gradually weakened as the solution pH was increased up to 7 from 11. On the one hand, the more alkaline the solution, the more negative charges accumulated on the surface of the MGO composite, which leads to the greater repulsion of the PMS negative ions and results in less PMS accumulating around the catalyst surface [48], and especially the pH value exceeding 9. On the other hand, the dominant form of CIP is the anion in strong alkali solutions, which also causes a repulsive force with the surface of the catalyst, and thereby consequently contributing to the tendency toward a reduction for both adsorption and degradation of CIP.

The effect of the concentration of MGO on CIP removal is revealed in Figure 5b, in which the concentration of PMS is controlled at 1.0 g/L. As can be seen, the final removal of CIP after 180 min treatment rose with the increase in the MGO dosage, and thereafter, it decreased as the dosage continued to increase. Specifically, as the concentration of MGO increased up to 2.0 g/L from 0 g/L, the CIP removal rate was significantly enhanced from 31.74% up to 94.11%. After that, it decreased to 93.04% for the MGO dosage of 3.0 g/L. This result indicated that moderately increasing the dosage of MGO was beneficial for the removal of CIP. On one hand, the adsorption of CIP on MGO could be improved by the increased MGO dosage. On the other, the increase in MGO could also provide additional active sites to promote the catalytic activation of PMS to generate oxidative radicals such as SO_4_^−^• and •OH, which would increase the degradation of CIP. However, the excessive MGO dosage decreased the removal of CIP, and this could be ascribed to the explosive generation of oxidative free radicals, which increased the invalid consumption of free radicals by other substances in a solution like Fe^2+^, as represented in the following reactions, Reactions (5) and (6) [13]:

(5) $$Fe^{2+}+SO_{4}{}^{-}•\to Fe^{3+}+SO_{4}{}^{2-}$$

(6) $$Fe^{2+}+•OH\to Fe^{3+}+OH^{-}$$

The contribution of PMS concentration for CIP removal was investigated (Figure 5c), where the MGO dosage was controlled as 2.0 g/L with the CIP initial concentration of 20 mg/L. As can be observed, the final removal rate of CIP after 180 min treatment increased with the addition of the PMS dosage, and thereafter, it declined with the addition of the dosage. Concretely, the CIP removal rate increased from 79.00 to 97.33% as the PMS dosage increased from 0.5 g/L to 2.0 g/L. Afterwards, it declined to 93.01% for the PMS dosage of 3.0 g/L. This result revealed that moderately increasing the amount of PMS contributed to the removal of CIP. SO_4_^−^• and other reactive species in the water increased as a result of the increasing concentration of PMS, which accelerated the degradation of CIP. Nevertheless, the excessive PMS dosage decreased the removal of CIP, which might be caused by the fact that the excessive generated free radicals would react with other substances in the solution, in particular with HSO_5_^−^, reacting with SO_4_^−^• and •OH to form SO_5_^−^•, being of less activity as well as a weak oxidative capacity [49]. This not only led to the unnecessary consumption of a large amount of oxygen-containing radicals but also reduced the effective utilization of PMS, resulting in a lower removal of CIP. The main reactions were as follows [45], Reactions (7)–(9):

(7) $$HSO_{5}{}^{-}+•OH\to SO_{5}{}^{-}•+H_{2}O$$

(8) $$HSO_{5}{}^{-}+SO_{4}{}^{-}•\to HSO_{4}{}^{-}+SO_{5}{}^{-}•$$

(9) $$SO_{4}{}^{-}•+SO_{4}{}^{-}•\to S_{2}O_{8}{}^{2-}$$

#### 3.2.3. Recyclability of MGO

**Figure 6:**
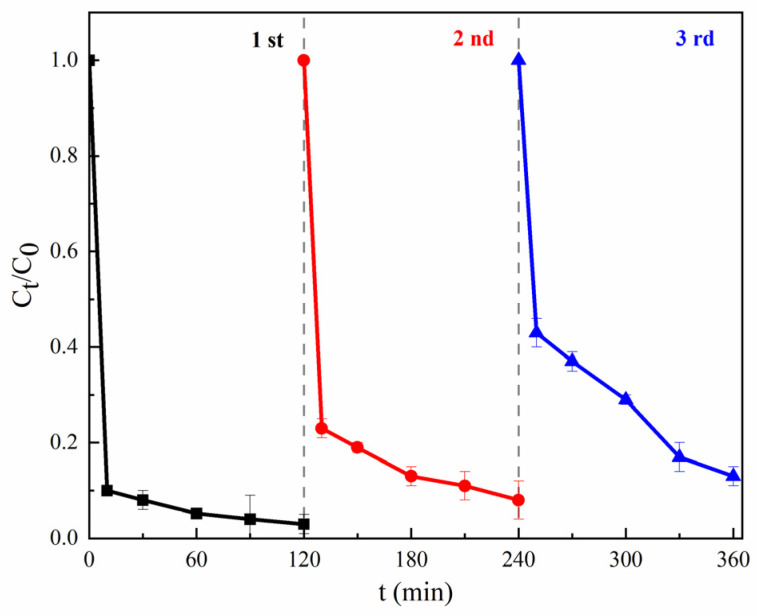
Stability and recyclability of MGO. Experimental conditions: [CIP] = 20 mg/L, [PMS] = 1.0 g/L, [MGO] = 0.5 g/L, pH = 7, T = 25 °C.

The stability and recyclability of the materials were considered as a vital condition for heterogeneous reactions. A magnetic test experiment (Figure S2) indicated that MGO displayed magnetic properties and allowed for magnetic separation. To evaluate the stability and recyclability of MGO, the used MGO was separated and collected magnetically, and washed repeatedly using a HCl solution (0.1 mol/L) and deionized water; after that, the regenerated MGO was ready for reuse. As shown in Figure 6, after 360 min treatments, the final removal rates of CIP were 96.5%, 92.2% and 87.0% for the first, second and third uses, respectively, which showed a gradually decreasing trend. This may be mainly due to the decrease in Fe^2+^ released into the solution, the incomplete elution of the adsorbed contaminants and the loss of the active functional groups on the surface of MGO. However, the catalytic performance of MGO was not significantly decreased and can meet the requirements of wastewater treatment, which may be accounted for regarding the protective effect of the multi-layer GO layers on Fe_3_O_4_, as shown in SEM and TEM images.

### 3.3. Mechanism of CIP Degradation by MGO-Activated PMS

**Figure 7:**
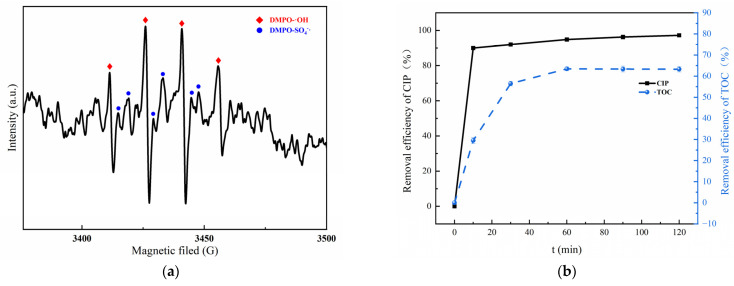
EPR spectra of DMPO–OH adduct and DMPO–SO_4_ in MGO/PMS system for 10 min (**a**); removal rate of CIP and TOC (**b**). Experimental conditions: 10 mL of deionized water, 2 mL of DMPO, pH = 7, T = 25 °C.

A consensus is that a number of oxidative radicals can be generated during the activation of PMS [50,51]. To verify the activation effect of MGO on PMS, an EPR detection was carried out utilizing DMPO as the spin trapping agent, which is presented in Figure 7a. As can be found, an obvious 1:2:2:1 four-peak DMPO-OH signal and a relatively weaker 1:1:1:1:1 six-peak DMPO-SO_4_ signal can be detected [52], which confirms the simultaneous presence of •OH and SO_4_^−^• in the MGO/PMS reaction system.

To investigate the mineralization ability of the MGO/PMS reaction system on organic pollutants, the CIP and TOC removal are compared in Figure 7b. As can be seen, after a 60 min reaction, the removal for CIP and TOC was 94.8% and 63.4%, correspondingly. What this result demonstrated is that both CIP and TOC could be effectively removed by the MGO/PMS reaction. Another fact was that the removal of TOC was significantly lower than that of CIP, which suggested the generation of organic intermediates during the degradation process.

**Figure 8:**
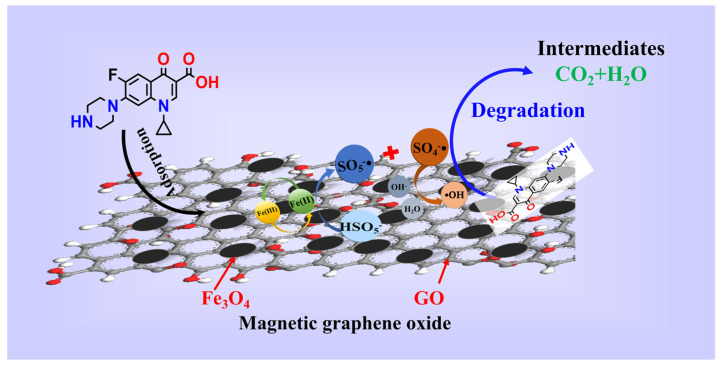
The mechanism of CIP degradation.

On the basis of the results of free radical trapping and CIP mineralization, as well as the combination of the FT-IR and XPS analysis, the mechanism of CIP degradation for MGO-activated PMS was speculated as follows. As shown in Figure 8, in the MGO/PMS system, in which both Fe_3_O_4_ and GO were involved in the reaction, the CIP degradation primarily proceeded with free radical processes. In one way, Fe^2+^ reacted with PMS, generating SO_4_^−^• and •OH, thereby oxidizing to Fe^3+^ with loss of electrons (Reactions (2) and (10)). Simultaneously, the Fe^3+^ obtained electron would initiate the regeneration of Fe^2+^ (Reaction (11)), thus forming a cyclic reaction chain that promoted free radical generation. For the other one, the modified GO might contain an abundance of free electrons, and the sp_2_ active carbon sites in GO, especially π electrons, exhibit a significantly higher electron transfer capacity, which induce the production of SO_4_^−^• with PMS (Reactions (12)–(15)) [44]. In addition, with the above reaction, in which some of the SO_4_^−^• will react with water molecules and OH^−^ in the solution to form a large amount of •OH (Reactions (16) and (17)) [52], the species and contents of the free radicals are affected dependent upon the pH of the solution, which is also in accordance with the results of the investigations in Section 3.2.2.

The reactive species such as SO_4_^−^• and •OH generated in the MGO/PMS system feature relatively high redox potentials, by which the CIP molecules may be attacked and transformed into smaller molecule intermediates that are eventually mineralized to CO_2_ and H_2_O with the redox reactions involving dehydroxylation, decarboxylation, etc. [44] (Reaction (18)).

(10) $$Fe^{2+}+HSO_{5}{}^{-}\to •OH+Fe^{3+}+SO_{4}{}^{2-}$$

(11) $$Fe^{3+}+HSO_{5}{}^{-}\to Fe^{2+}+SO_{5}{}^{-}•+H^{+}$$

(12) $$C-\pi +HSO_{5}{}^{-}\to SO_{4}{}^{-}•+C-\pi^{+}+OH^{-}$$

(13) $$C-\pi^{+}+HSO_{5}{}^{-}\to SO_{4}{}^{-}•+C-\pi +H^{+}$$

(14) $$C=C=O+HSO_{5}{}^{-}\to SO_{4}{}^{-}•+C=C=O^{+}+OH^{-}$$

(15) $$C=C=O^{+}+HSO_{5}{}^{-}\to SO_{4}{}^{-}•+C=C=O+H^{+}$$

(16) $$SO_{4}{}^{-}•+H_{2}O\to •OH+SO_{4}{}^{2-}$$

(17) $$SO_{4}{}^{-}•+OH^{-}\to •OH+H^{+}$$

(18) $$SO_{4}{}^{-}•/•OH+CIP\to \mathrm{Intermediates}+{\mathrm{CO}}_{2}+H_{2}O$$

**Figure 9:**
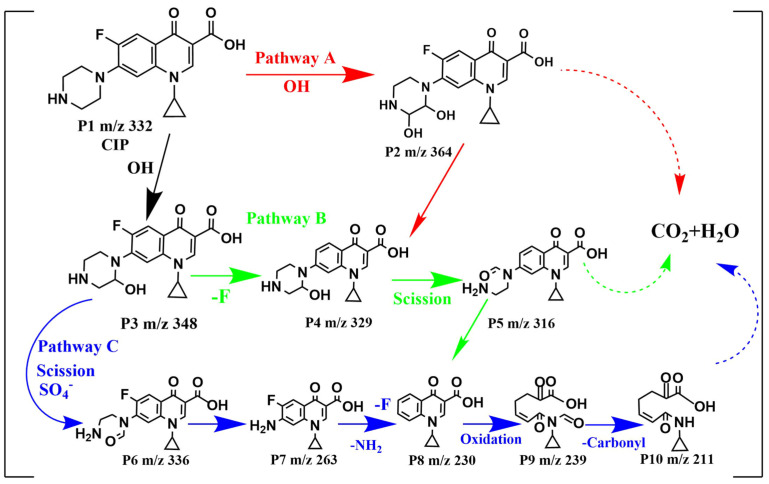
Proposed degradation pathways for CIP by MGO-activated PMS.

The intermediates were generated from the reaction between CIP and oxidative radicals. To identify the intermediates, the LC-MS spectrum CIP solutions before and after 120 min treatment were compared in Figure S3a,b, and the corresponding intermediates speculated on based on the mass-to-charge ratios are listed in Table S1. On the basis of the detection of intermediates and oxidized radicals, the degradation mechanism of CIP in the MGO/PMS reaction system could be proposed as shown in Figure 9, which mainly has three degradation pathways.

For pathway A, the piperazine ring of CIP attacked with •OH formed either P2 (*m*/*z* = 364) or P3 (*m*/*z* = 348). In pathway B, P2 (*m*/*z* = 348) was directly defluorinated to generate P4 (*m*/*z* = 329), which formed P5 (*m*/*z* = 316) with the direct piperazine ring opening and ‘-CH3′ loss. Pathway C initiated with an oxidation reaction process in which an electron in the piperazine group was substituted by SO_4_^−^•, followed by what seemed to be the side chain of the piperazine ring of P3 (*m*/*z* = 348) fracturing and opening for the formation of P6 (*m*/*z* = 336) [53,54]. Following further oxidation, the piperazine ring was completely removed to generate P7 (*m*/*z* = 263), which was transformed to P8 (*m*/*z* = 230) as a direct defluorination ion and amino group. As the quinoline ring of P8 continued to be opened by oxidation and a decarbonylation reaction occurred, P9 (*m*/*z* = 239) and P10 (*m*/*z* = 211) were successively generated. After that, P2, P5 and P10 produced from pathway A, B and C, respectively, would be further degraded into final products (small organic compounds, CO_2_ and H_2_O, etc.). In degrading CIP, the quinolone ring in the CIP molecule appears to be the weakest in oxidability, followed by the benzene ring, while the piperazine ring shows the highest oxidability [55]. Consequently, pathways B and C were the dominant degradation pathways for CIP, while pathway A was the secondary one.

## 4. Conclusions

In this study, an MGO composite was synthesized by the ultrasonic chemical precipitation method and characterized with SEM, TEM, BET, XRD, FT-IR and XPS in terms of morphological features, structure, functional groups and elemental groups. As compared with the degradation of CIP by PMS alone, GO/PMS and Fe_3_O_4_/PMS, the MGO/PMS system exhibited the better performance of the CIP removal, which could be attributed to the catalytic activation of PMS with MGO. For the solution with an initial CIP concentration of 20 mg/L, a maximum removal could reach up to 96.5% at the optimum reaction conditions, which corresponds to a TOC removal rate of 63.4%. A kinetic model of pseudo-secondary adsorption indicated a dynamic process influenced by chemisorption. After three cycles of reuse of MGO, the CIP removal rate can be maintained at 87.0%, which demonstrates the stability and recyclability of MGO. The EPR results confirmed that oxidative radicals generated in the MGO/PMS system, particularly •OH and SO_4_^−^• play a dominant role for CIP degradation. The TOC removal rate and LC-MS spectrum indicated that CIP was partially degraded into organic intermediates, and the degradation mechanism of CIP was proposed as a process with three degradation pathways. It is thus assumed based on our investigation results that MGO serves as a potential catalyst for PMS activation, which presents promising research and application prospects in the field of wastewater treatment.

## Figures and Tables

**Table 1:** The Surface Area, Pore Volume and Pore Size of Fe_3_O_4_, GO and MGO.

	Surface Area (m^2^/g)	Pore Volume (cm^3^/g)	Pore Size (nm)
Fe_3_O_4_	70	0.2796	158.00
GO	140	0.3337	94.49
MGO	163	0.3921	95.48

**Table 2:** Elemental analysis of MGO.

Material	C (%)	O (%)	Fe (%)	Total (%)	C/O
MGO	35.60	46.82	17.58	100.00	0.76

## Data Availability

Data are available from the corresponding author by request.

## Supplementary Materials

The following supporting information can be downloaded at: https://www.mdpi.com/article/10.3390/toxics11121016/s1, Figure S1: Pseudo-first-order degradation kinetic model (a), Pseudo-sencond-order degradation kinetic model (b) and Pseudo-first-order adsorption kinetic model (c); Figure S2: Magnetic properties test of MGO. (magnetic separation of solid states (a), magnetic separation of MGO in aqueous solution ((b): 1 min; (c): 30 min)); Figure S3: LC-MS detection results for CIP before treatment (a) and after 120 min of treatment (b); Table S1: Analysis of intermediates from degradation of CIP.
