# Supplementary material for: Degradation of Ciprofloxacin in Water by Magnetic-Graphene-Oxide-Activated Peroxymonosulfate
Source: Toxics. 2023 Dec 13;11(12):1016. doi: 10.3390/toxics11121016 (PMC10747872; doi:10.3390/toxics11121016)
Supplement: Supplementary file 1 [file toxics-11-01016-s001.zip › toxics-2741982-supplementary.pdf]

Supplementary Materials: Degradation of Ciprofloxacin in Water by Magnetic-Graphene-Oxide-Activated Peroxymonosulfate

Xiaoping Wang, Yulan Li, Jiayuan Qin, Ping Pan, Tianqing Shao, Xue Long and Debin Jiang

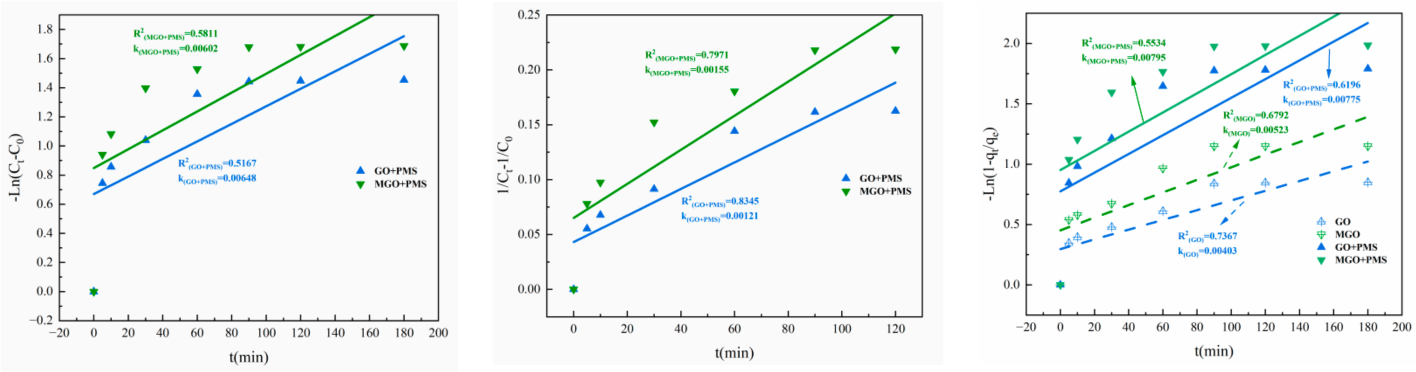

(a) (b) (c)

Figure S1. Pseudo-first-order degradation kinetic model (a), Pseudo-sencond-order degradation kinetic model (b) and Pseudo-first-order adsorption kinetic model (c).

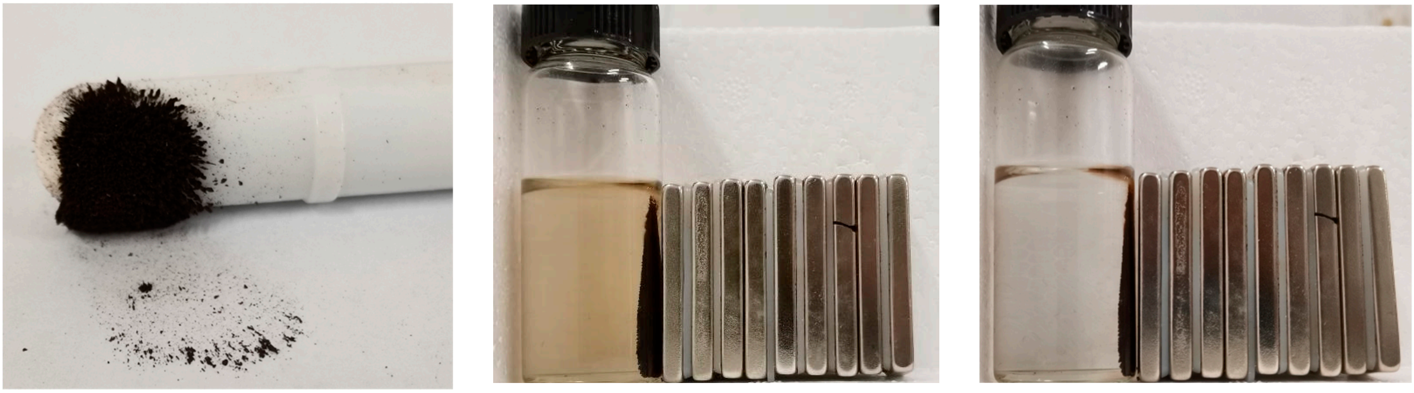

(a) (b) (c)

Figure S2. Magnetic properties test of MGO. (magnetic separation of solid states (a), magnetic separation of MGO in aqueous solution ((b): 1 min; (c): 30 min)).

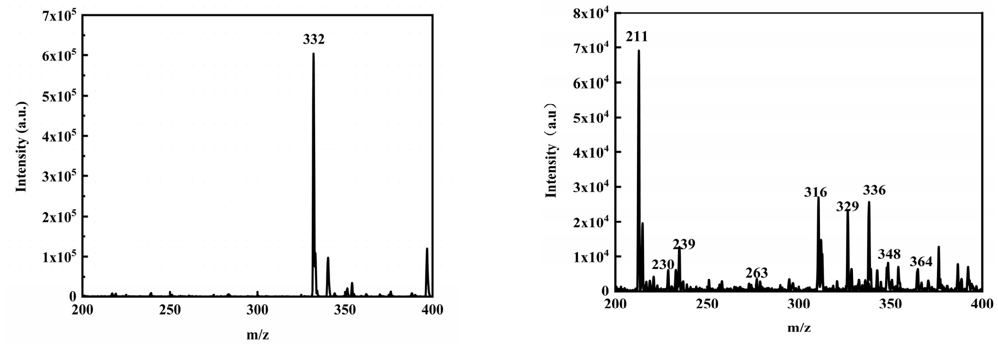

(a) (b)

Figure S3. LC-MS detection results for CIP before treatment (a) and after 120 min of treatment (b).

Table S1. Analysis of intermediates from degradation of CIP.

Serial No.	m/z	Molecular formula	Structural formula
1	332	$C_{17}H_{18}FN_3O_3$	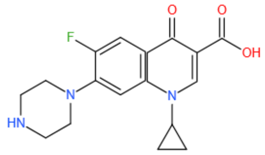
2	364	$C_{17}H_{18}FN_3O_5$	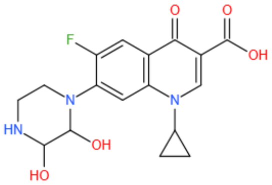
3	348	$C_{17}H_{18}FN_3O_4$	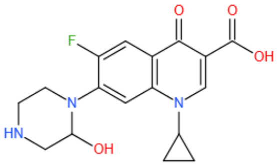
4	336	$C_{16}H_{16}FN_3O_4$	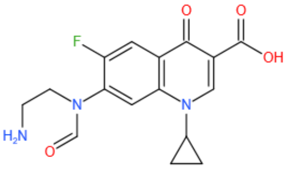
5	329	$C_{17}H_{18}N_3O_4$	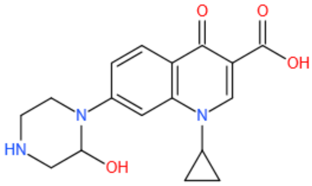
6	316	$C_{16}H_{16}N_3O_5$	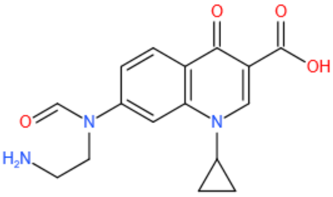
7	263	$C_{13}H_{11}FN_2O_3$	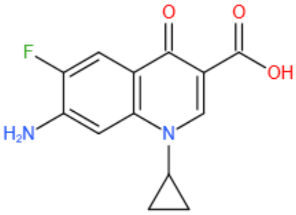
8	239	$C_{11}H_{13}NO_5$	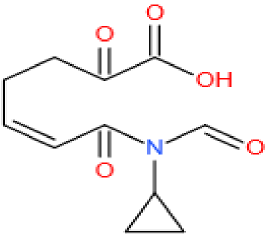

9	230	$C_{13}H_{11}O_3$	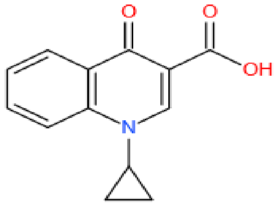
10	211	$C_{10}H_{13}NO_4$	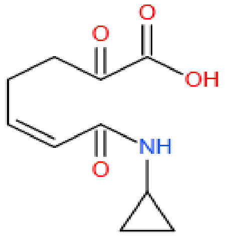
